# Incidentally Detected Congenital Bronchial Atresia in Late Adulthood: A Case Report and Imaging Review

**DOI:** 10.7759/cureus.112801

**Published:** 2026-07-16

**Authors:** Abdelkader Sqalli Houssaini, Ola Messaoud, Zaynab Iraqi Houssaini, Omar El Aoufir, Laila Jroundi

**Affiliations:** 1 Radiology, Ibn Sina Hospital, Mohammed V University, Rabat, MAR

**Keywords:** bronchial atresia, bronchocele, chest ct, congenital bronchial anomaly, focal hyperlucency, mucoid impaction

## Abstract

Bronchial atresia is a rare congenital anomaly characterized by focal interruption of a bronchus, typically associated with distal mucoid impaction and hyperinflation of the corresponding lung parenchyma. It is usually identified incidentally in children or young adults, whereas diagnosis in late adulthood is uncommon. We report the case of a 59-year-old man who presented with chronic cough and dyspnea. Chest computed tomography (CT), performed before and after intravenous contrast administration, revealed focal unilateral hyperlucency involving the posterior segment of the right upper lobe, associated with a moniliform bronchocele containing mucoid impaction and interruption of the proximal continuity of a dorsal subsegmental bronchus with the segmental bronchus and the central tracheobronchial tree. No enhancing endobronchial lesion, suspicious pulmonary mass, post-obstructive consolidation, or significant lymphadenopathy was identified. These findings were highly suggestive of congenital bronchial atresia. The patient was referred for pulmonology evaluation. In the absence of superinfection, significant functional impairment, or persistent diagnostic uncertainty, conservative management with clinical follow-up was recommended. The patient remained clinically stable during five months of follow-up, with no worsening of symptoms or recurrent respiratory infection.

This case highlights the key role of CT in establishing the diagnosis of bronchial atresia in adulthood and in differentiating it from other causes of focal hyperlucency and bronchial obstruction.

## Introduction

Bronchial atresia is an uncommon congenital malformation characterized by focal interruption of a lobar, segmental, or subsegmental bronchus, usually associated with distal mucoid impaction and hyperinflation of the involved lung parenchyma [1-3]. It is most often discovered incidentally, especially in children and young adults, because many individuals remain asymptomatic or present with only mild, nonspecific respiratory symptoms [1,4,5].

Computed tomography (CT) plays a central role in diagnosis by demonstrating the typical association of bronchocele, distal hyperlucency, decreased vascularity, and absence of normal proximal communication with the central tracheobronchial tree [2,3,6,7]. Recognizing this characteristic imaging pattern is important to avoid confusion with other causes of focal hyperlucency or bronchial obstruction, particularly acquired obstruction in adult patients [4,5,8].

This case should be documented because late-adulthood recognition of congenital bronchial atresia is uncommon and may raise concern for acquired bronchial obstruction. It provides an opportunity to emphasize the CT features that support the diagnosis, the radiological exclusion of acquired obstruction, and the rationale for conservative management when complications are absent.

## Case presentation

In October 2025, a 59-year-old man with no significant past medical history presented for evaluation of a persistent cough, which was the primary complaint at presentation. The cough had been present for four years and was moderate, associated with mild progressive exertional dyspnea. The persistence of symptoms and gradual worsening of exertional dyspnea prompted the current consultation. He reported no hemoptysis, fever, weight loss, chest pain, or recurrent respiratory infections. Physical examination was unremarkable. Pulmonary function testing was not available at the time of initial evaluation.

Chest CT was performed before and after intravenous contrast administration, with lung-window and mediastinal-window reconstructions.

**Figure 1:**
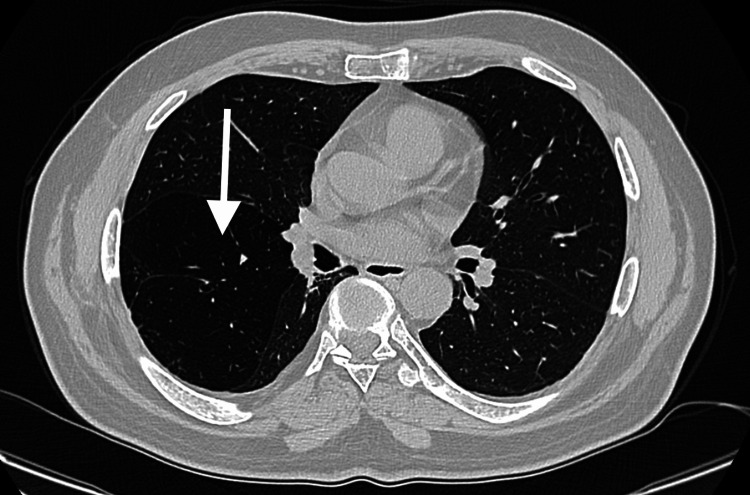
Focal hyperlucency in the right upper lobe Axial chest CT image in lung window showing focal hyperlucency and overdistension involving the posterior segment of the right upper lobe (white arrow).

**Figure 2:**
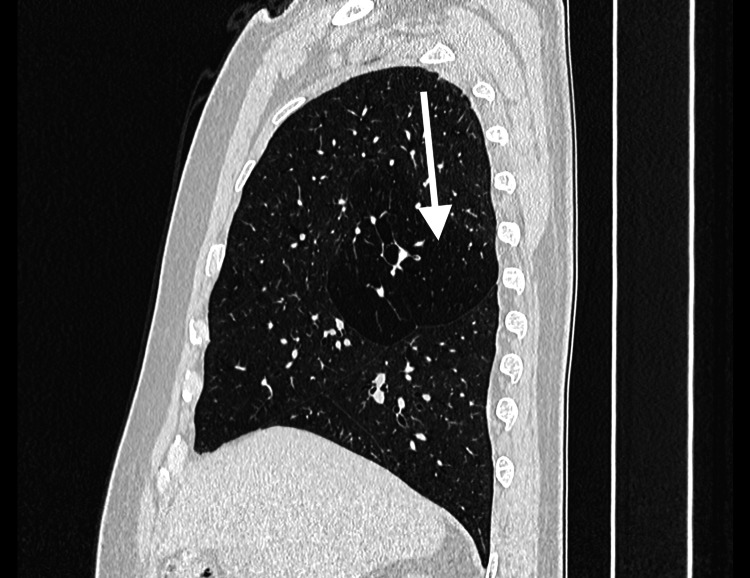
Focal hyperlucency confirmed in sagittal reconstruction Sagittal reconstruction confirming the extent of hyperlucency within the same segment (white arrow).

**Figure 3:**
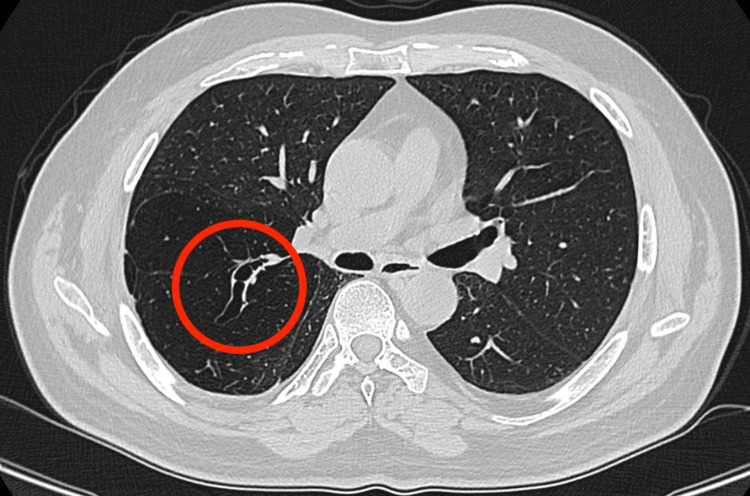
Bronchocele with mucoid impaction Axial chest CT image in lung window demonstrating a branching tubular opacity consistent with a bronchocele containing mucoid impaction within the affected subsegmental bronchus (red circle).

**Figure 4:**
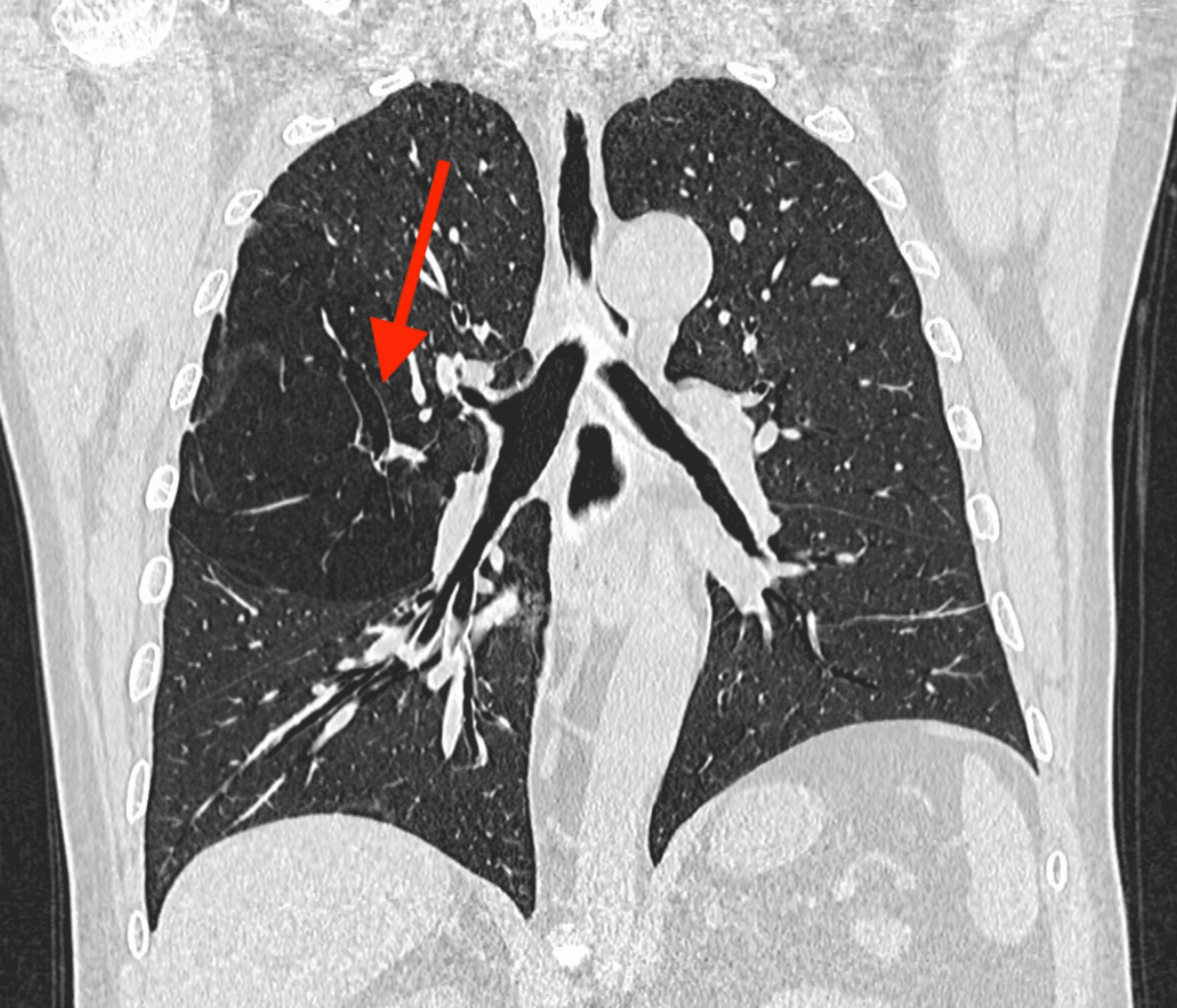
Demonstration of bronchial interruption on coronal reconstruction Coronal reconstruction clearly demonstrating the absence of continuity between the affected subsegmental bronchus and the central tracheobronchial tree (red arrow).

Lung window images demonstrated focal unilateral hyperlucency involving part of the posterior segment of the right upper lobe, with associated distension (Figures 1-2). Within this area, a moniliform bronchial dilatation containing mucoid impaction was identified (Figure 3). There was an interruption of the proximal continuity between the dorsal subsegmental bronchus and the dorsal segmental bronchus of the right upper lobe, with no visible communication with the central tracheobronchial tree (Figure 4). 

**Figure 5:**
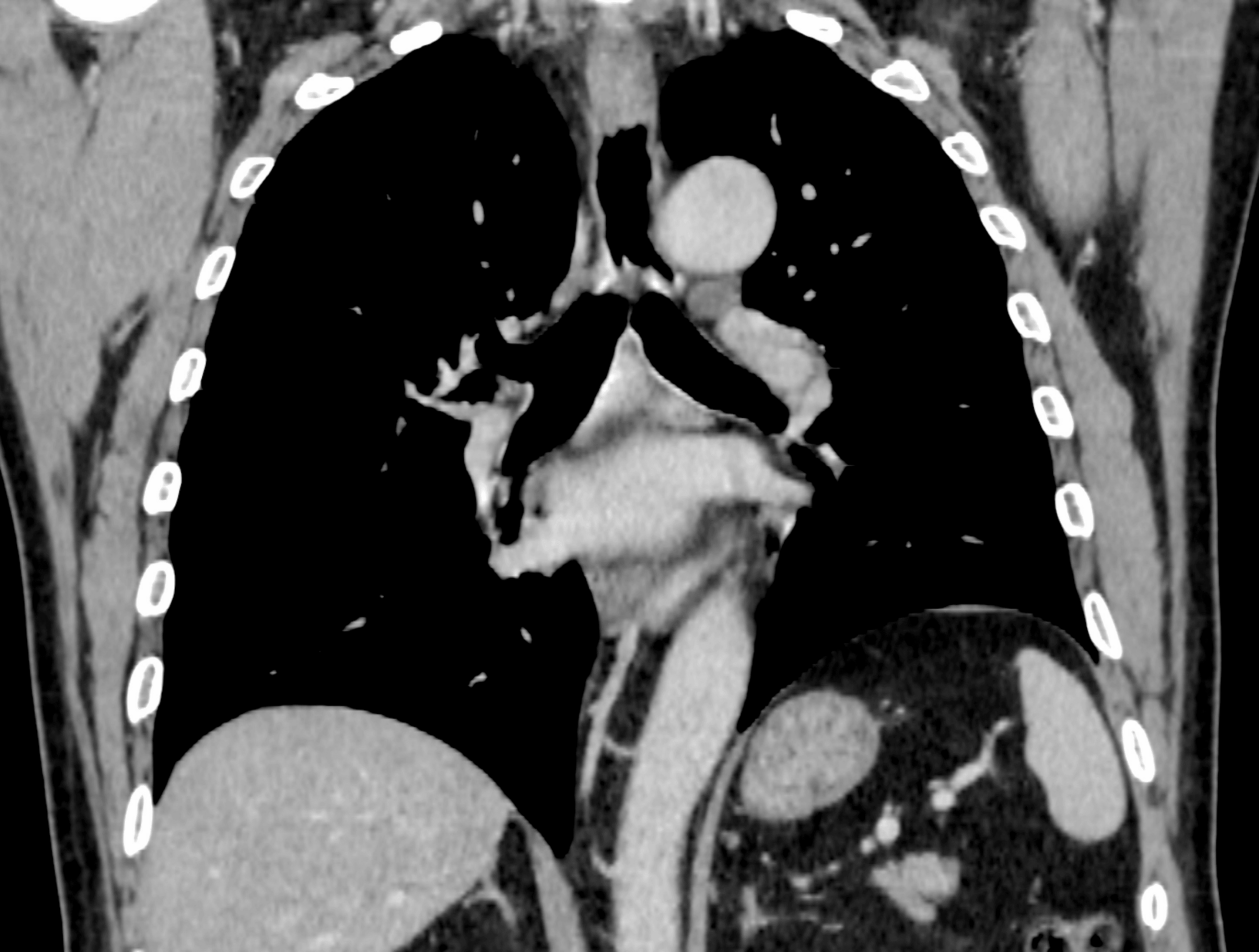
Coronal post-contrast CT showing no acquired obstructive lesion Coronal contrast-enhanced chest CT reconstruction demonstrating the absence of an enhancing endobronchial lesion, suspicious pulmonary mass, post-obstructive consolidation, and significant mediastinal or hilar lymphadenopathy.

Additional lung-window findings included mild linear atelectatic bands involving the lingula, middle lobe, and left lower lobe, interpreted as nonspecific incidental findings unrelated to the right upper lobe bronchial atresia. No ground-glass opacity, parenchymal consolidation, or suspicious pulmonary nodule was identified. Coronal post-contrast mediastinal-window images showed no enhancing endobronchial lesion, suspicious pulmonary mass, significant mediastinal, hilar, or axillary lymphadenopathy, pleural or pericardial effusion, or other finding suggestive of acquired bronchial obstruction (Figure 5).

Based on the characteristic CT findings and the absence of radiological features suggesting acquired bronchial obstruction, the diagnosis of congenital bronchial atresia involving a right upper lobe dorsal subsegmental branch, associated with distal bronchocele, was considered highly likely.

The patient was referred for pulmonology evaluation. No specific treatment directed at bronchial atresia was initiated because there was no evidence of superinfection, no suspicious obstructive lesion, and no significant functional impairment. Symptomatic treatment was not considered necessary at that stage because the symptoms remained mild, and there was no indication for antibiotics, bronchodilator therapy, or surgical intervention.

The planned follow-up strategy included pulmonology reassessment, monitoring of cough and dyspnea, and clinical surveillance for recurrent infection or symptom progression. Repeat chest imaging and pulmonary function testing were to be considered if symptoms worsened or if diagnostic uncertainty persisted. The patient remained clinically stable during five months of follow-up, with no worsening of cough or dyspnea, no fever, no hemoptysis, and no recurrent respiratory infection.

## Discussion

Bronchial atresia is a rare congenital anomaly resulting from focal interruption of a lobar, segmental, or subsegmental bronchus. Distal to the atretic segment, retained mucus accumulates within the obstructed bronchus, producing a bronchocele, while collateral ventilation through the pores of Kohn and channels of Lambert leads to air trapping and hyperinflation of the corresponding lung parenchyma [1-3]. Thus, although the affected bronchus is proximally interrupted, the distal lung parenchyma may remain aerated through collateral pathways, explaining why bronchial atresia is typically associated with focal hyperlucency rather than collapse. This pathophysiologic mechanism explains the characteristic imaging pattern of the disease.

Although bronchial atresia is congenital, it is often discovered incidentally because many patients remain asymptomatic or present only mild, nonspecific respiratory symptoms [4,5]. When symptomatic, patients may report chronic cough, dyspnea, or recurrent localized infections [1-3]. Diagnosis in late adulthood is uncommon, which makes the present case noteworthy. In our patient, the anomaly was identified at 59 years of age during the evaluation of chronic cough and dyspnea.

CT is the imaging modality of choice and may allow a confident noninvasive diagnosis when the classic imaging pattern is present and acquired bronchial obstruction has been carefully excluded [6,7]. The classic CT triad includes a branching or tubular bronchocele, absence of normal proximal communication with the central tracheobronchial tree, and distal hyperlucency related to air trapping, frequently accompanied by reduced vascularity in the involved pulmonary territory [4,7]. Matsushima et al. reported that CT consistently demonstrated mucocele formation, proximal bronchial occlusion, and emphysematous change in the distal lung, highlighting the central role of CT in establishing the diagnosis [6]. In the present case, the combination of a moniliform mucus-filled bronchial dilatation, focal hyperlucency in the posterior segment of the right upper lobe, and interruption of proximal bronchial continuity was highly suggestive of bronchial atresia.

From an anatomic perspective, bronchial atresia most frequently affects the upper lobes, particularly the left upper lobe, although right upper lobe involvement is well recognized [2,5,6]. The present case is therefore consistent with the known distribution of the disease, while remaining of educational interest because of its incidental detection in late adulthood.

The main differential diagnoses include other causes of focal hyperlucency and mucus-filled bronchi, such as congenital lobar overinflation, Swyer-James-MacLeod syndrome, post-infectious bronchiolar obliteration, bronchogenic cyst, allergic bronchopulmonary aspergillosis, and acquired endobronchial obstruction due to tumor or foreign body [5-8]. In adult patients, one of the main diagnostic challenges is to differentiate congenital bronchial atresia from acquired bronchial obstruction. In this regard, CT is especially valuable because bronchial atresia typically appears as a blind-ending mucus-filled bronchus without an enhancing obstructive lesion, whereas acquired obstruction is more likely to be associated with a visible endobronchial mass or secondary post-obstructive changes [4,5,8]. In our case, the absence of a suspicious obstructive lesion and the characteristic bronchocele-hyperlucency pattern strongly supported the diagnosis of congenital bronchial atresia. Bronchoscopy may be useful when CT findings are equivocal or when an acquired obstructive lesion remains clinically suspected. In the present case, bronchoscopy was not performed because no obstructive lesion was identified on contrast-enhanced CT, and the patient remained clinically stable during follow-up.

Management is usually conservative in asymptomatic or mildly symptomatic patients, while surgical treatment is generally reserved for recurrent infection, significant symptoms, progressive destruction of the affected lung parenchyma, or persistent diagnostic uncertainty [1,3]. In the present case, the patient was referred for pulmonology evaluation. In the absence of superinfection, significant functional impairment, suspicious obstructive lesion, or persistent diagnostic uncertainty, conservative management with clinical follow-up was recommended. No specific treatment directed at bronchial atresia was initiated because there was no indication for antibiotics, bronchodilator therapy, or surgical intervention. The patient remained clinically stable during five months of follow-up, with no worsening of respiratory symptoms and no recurrent infection. This evolution is consistent with the generally benign course reported in uncomplicated cases. This report has some limitations. Bronchoscopy was not performed, and pulmonary function testing was not available at the time of initial evaluation. However, contrast-enhanced CT did not show any suspicious endobronchial lesion or other feature suggestive of acquired bronchial obstruction, and the patient remained clinically stable during follow-up.

In summary, this case emphasizes the importance of recognizing the typical CT features of bronchial atresia in adults. Even when diagnosed late, the association of bronchocele, focal hyperlucency, and interruption of proximal bronchial continuity is highly suggestive and may help avoid unnecessary invasive investigations or overtreatment [1-5].

## Conclusions

This case highlights the importance of recognizing the classic imaging pattern of congenital bronchial atresia, even when discovered in late adulthood. The association of bronchocele, focal hyperlucency, and interruption of proximal bronchial continuity on CT is highly suggestive of the diagnosis, particularly after acquired bronchial obstruction has been carefully excluded. In adult patients, careful radiological assessment is essential to avoid misdiagnosis with acquired obstructive conditions. A conservative approach with clinical follow-up remains appropriate in the absence of infection, significant functional impairment, or clinical and radiological features suggesting acquired bronchial obstruction.
